# Efficacy and safety of a low-sodium diet and spironolactone in patients with stage 1-3a chronic kidney disease: a pilot study

**DOI:** 10.1186/s12882-022-02711-z

**Published:** 2022-03-05

**Authors:** Hongmei Zhang, Bin Zhu, Liyang Chang, Xingxing Ye, Rongrong Tian, Luchen He, Dongrong Yu, Hongyu Chen, Yongjun Wang

**Affiliations:** Department of Nephrology, Hangzhou TCM Hospital Affiliated to Zhejiang Chinese Medical University, 453 Tiyuchang Road, Hangzhou, 310007 China

**Keywords:** Low-sodium diet, Mineralocorticoid receptor antagonist, Chronic kidney disease, Spironolactone, Safety

## Abstract

**Background:**

Excessive salt intake is associated with the deterioration of chronic kidney disease (CKD). Aldosterone is also known as an independent risk factor for kidney injury. Dietary sodium intake acts as a main stimulator in aldosterone-mediated kidney injury. Hence, this study aimed to further investigate the renal protective effects and safety of a low-sodium diet in combination with spironolactone (SPL) in stage 1-3a CKD.

**Methods:**

This single-center, SPL-blinded randomized controlled trial recruited patients with stage 1-3a CKD, randomized into three groups, low-sodium (3 g/d salt) + placebo, medium-sodium (5 g/d salt) + SPL, and low-sodium (3 g/d salt) + SPL. Patients received 12 weeks of intervention. The primary and secondary endpoints were 24-h urine protein and estimated glomerular filtration rate (eGFR) at the end of the intervention, respectively.

**Results:**

A total of 74 patients were analyzed eventually. Significantly decreased 24-h urine protein was found in all three groups, from 0.37 to 0.23 g/d (*P* = 0.004) in the low-sodium+placebo group, from 0.44 to 0.29 g/d (*P* = 0.020) in the medium-sodium+SPL group, and from 0.35 to 0.31 g/d (*P* = 0.013) in the low-sodium +SPL group. There were no significant differences among the three groups in 24-h urine protein amount change after intervention from pre-treatment values (*P* = 0.760, ITT set). The results of the 24-h urine protein by using PP set analysis was similar to the ITT set. No significant differences in eGFR, nutritional, metabolic, inflammatory, and other biomarkers were observed across all three groups (*P* > 0.05). No safety signal was observed.

**Conclusion:**

No additional benefit was observed when SPL was prescribed to patients already on a low-sodium diet (3.0 g/d). Still, small doses of SPL may benefit patients with poor sodium restriction. A combination of short-term low-dose SPL and ARB is safe for patients with stage 1-3a CKD, but blood potassium must be regularly monitored.

**Trial registration:**

Name of the registry: Chinese clinical trial registry.

Trial registration number: ChiCTR1900026991.

Date of registration: Retrospectively registered 28 October 2019.

URL of trial registry record: http://www.chictr.org.cn/searchproj.aspx?title=&officialname=&subjectid=&secondaryid=&applier=&studyleader=ðicalcommitteesanction=&spo

**Supplementary Information:**

The online version contains supplementary material available at 10.1186/s12882-022-02711-z.

## Background

Chronic kidney disease (CKD) incidence is growing worldwide. The USRDS in 2018 revealed that CKD prevalence in adults reached 14.8% in 2013–2016 [1]. An epidemiological survey in China in 2012 indicated a CKD prevalence of 10.8% in adults over 18 years old [2]. For patients with CKD, early intervention is of great importance in controlling CKD progression and reducing mortality.

High salt intake is closely associated with the progression of CKD. When the urine sodium-to-creatinine ratio increases by 100 mmol/L, the risk of CKD developing into end-stage renal disease (ESRD) increases by 1.61 times [3, 4]. High salt intake leads to renal impairment in various ways, including increasing transforming growth factor (TGF)-β1 production and enhancing oxidative stress and inflammatory response in the kidney [5–7]. Salt restriction reduces the effects of the above adverse factors, protecting the kidney and enhancing the antiproteinuric and antihypertensive effects of the renin-angiotensin-aldosterone system (RAAS) antagonists such as angiotensin-converting enzyme inhibitors (ACEIs) and angiotensin II receptor blockers (ARBs) [8, 9]. Heeg et al. [9] found that the effectiveness of the ACEI lisinopril in reducing albuminuria greatly depends on dietary sodium intake. The ACEI could not decrease albuminuria when sodium intake from food increased from 50 to 200 mmol/d, while the decreasing effect of the ACEI on albuminuria recovered accordingly when sodium intake was reduced back to 50 mmol/d. Vogt et al. [10] showed that proteinuria was reduced by 30% with losartan monotherapy alone. The reduction increased to 55% with the addition of salt restriction and to 56% with the addition of hydrochlorothiazide (HCT). The enhancing effects of salt restriction on ARB activity appeared similar to the addition of diuretics. Slagman et al. [11] reported that moderate dietary sodium restriction is more effective than the maximal dose of angiotensin receptor blocker in controlling proteinuria and blood pressure in patients with renal disease on a maximal dose of ACEI.

Aldosterone is a steroid hormone with mineralocorticoid activity. Historically, aldosterone has been shown to act mainly on the distal convoluted tubules of the kidney, regulating extracellular fluid capacity and potassium metabolism. However, in the past 20 years, studies have revealed an extensive role played by aldosterone [12–16]. Mounting evidence has been observed that aldosterone could affect the heart, blood vessels, the central nervous system, and the kidney, promoting vascular remodeling, collagen formation, and endothelial dysfunction [15, 16]. These interactions play important roles in the pathophysiology of progressive renal dysfunction. Moreover, aldosterone/mineralocorticoid receptor (MR) could also damage podocytes. The enhanced MR effect is closely associated with protein leakage in the kidney [17–20], leading to CKD progression. With these new insights, mineralocorticoid receptor antagonists (MRAs) as a new treatment strategy are of particular interest. The discovery of the “aldosterone escape” phenomenon has made MRAs even more attractive for the treatment of CKD [21]. Multiple studies have evaluated the effects of MRAs on blood pressure control and proteinuria reduction, as well as their possible role in delaying the progression of CKD [14, 22, 23].

Aldosterone has been recognized as an independent risk factor mediating renal injury. However, another important parameter, sodium intake, cannot be ignored. More than 70 years ago, a pioneering study by Hans Selye showed that when desoxycorticosterone acetate (DOCA) is used in a rodent model of partial nephrectomy, improper salt intake (3% saltwater) is required for inducing significant vasculitis changes in the heart and kidney [24]. A study of Dahl salt-sensitive rats also showed that high salt could induce oxidative stress and promote MR activation in the kidney [20]. These studies helped to understand the pathogenesis of MR-induced renal injury and establish reasonable treatment plans in CKD. Relevant research data also demonstrated that salt intake could affect the balance of beneficial and adverse effects of aldosterone [25]. In case of improper salt intake, acute administration of aldosterone reduces the levels of phosphorylated extracellular nitric oxide synthase (eNOS; vascular protection), increases the amounts of phosphorylated extracellular signal-regulated kinases 1 and 2 (ERK1/2) and protein kinase C (adverse to blood vessels). Conversely, salt intake reduction increases phosphorylated eNOS levels, reduces ERK1/2 and protein kinase C amounts, and minimizes or reverses the response to acute aldosterone administration. Plasma aldosterone levels unsuitable for intake of diet salt would amplify the above phenomenon. Hattori et al. [26] found that under low-salt conditions, MRA can completely suppress the expression of RAS-related genes in the myocardium of rats, alleviate myocardial oxidative stress and inflammatory response, and delay myocardial hypertrophy and fibrosis; meanwhile, MRA is only partly effective under high-salt conditions.

Based on the above, salt restriction is not only beneficial for renal protection but also can enhance the proteinuria-reducing effect of ACEI/ARB while alleviating aldosterone-associated renal damage. So far, the main treatment delaying CKD progression and reducing the risk of end-stage renal disease is RAAS antagonists, including ACEI and ARB medications, as well as direct renin inhibitors. These medications are the current standard treatment options for CKD patients with proteinuria [27–31]. However, these medications only decrease the risk of proteinuria and ESRD by 20–30%. Additional new therapies are needed given the huge cost burden of CKD as well as the associated physiological and psychological damage to patients [32, 33]. Therefore, this study aims to investigate the renal protective effects and safety of a low-sodium diet combined with an MRA, spironolactone (SPL), in patients with stage 1-3a CKD.

## Methods

### Participants

Patients with stage 1-3a CKD were consecutively recruited from the outpatient Department of Nephrology in Hangzhou Hospital of Traditional Chinese Medicine from September 2014 to April 2017. According to K-DOQI guidelines [3, 4], the diagnostic criteria for CKD include: 1) renal injury (abnormal renal structure or function) for ≥3 months, with or without glomerular filtration rate (GFR) decrease; 2) GFR < 60 ml/(min·1.73 m^2^) for ≥3 months, with or without evidence of renal injury.

Inclusion criteria were 1) primary chronic glomerular disease with positive urinary protein or albumin, 2) estimated GFR (eGFR) ≥ 45 ml/min/1.73 m^2^, 3) aged 18–70 years, 4) no clinical evidence of acute injury, and 5) no mental disorder and ability to cooperate.

Exclusion criteria were 1) treatment with glucocorticoids, immunosuppressants, or nonsteroidal anti-inflammatory medications (NSAIDs) for more than one week in the past three months, 2) serious primary diseases affecting organs such as the heart, brain, lung, liver, or hematopoietic system, 3) malignant tumors, tuberculosis, and/or other acute infectious diseases, 4) pregnancy, lactation or pregnancy planning in the near future in women, 5) hypersensitivity to MRAs such as SPL, and 6) participation in other medication-based clinical trials.

### Study design

This was a single-center, double-blinded (SPL-blinded), randomized controlled trial. Patients were randomized into three groups and received the assigned intervention for 12 weeks. The random numbers were produced by means of a random number table in advance and were kept in an opaque envelope. After the intervention, blinding was uncovered. The current study was approved by the ethics committee of Hangzhou Hospital of Traditional Chinese Medicine (2013LL065), and written informed consent was obtained from the patients.

### Grouping and treatment

Eligible patients were randomly assigned to three groups before the run-in period started. (1) Low-sodium+placebo group. Patients underwent strict control of daily dietary salt intake. The salt bag (3 g/bag) and salt control spoon were provided, and the salt bag was replaced at the return visit. The appearance, size, weight, smell, and color of the placebo (Zhejiang Conba Pharmaceutical Co., Ltd) were the same as those of SPL, and it was administered orally at 40 mg the first week and 20 mg thereafter, once a day, 30 min after breakfast. (2) Medium-sodium+SPL group. The salt bag (5 g/bag) and salt control spoon were provided, and SPL (20 mg tablets; Hangzhou Minsheng Pharmaceutical Co., Ltd) was taken. SPL was administered orally at 40 mg the first week and 20 mg thereafter, once a day, 30 min after breakfast. (3) Low-sodium+SPL group. Patients underwent strict control of daily dietary salt intake. Supplies and medications such as SPL were provided, and the salt bag was replaced at each visit. The intervention lasted for 12 weeks.

In the run-in period (0–4 weeks), patients received training in optimal blood pressure and blood lipid control, salt intake assessment, and high-quality dietary choice with proper protein intake. Basic treatment during this period included the following: 1) Dietary guidance according to Chinese expert consensus on protein nutrition therapy for chronic kidney disease: dietary protein intake is 0.8 g/kg/d-1.0 g/kg/d with 50% high biological value protein, and dietary energy intake is 30–35 kcal/kg/d, and education of adult patients for low-fat diet during treatment. 2) Blood pressure control: the ARB dose was stable for more than 3 months at the time of enrollment and maintained throughout the study. Among the patients, 79.7% used ARBs (Irbesartan at 75–150 mg or Cozaar at 50–100 mg), 10.8% used calcium channel blocker (CCB), and 1 patient used β blocker. CCB and other antihypertensive medications were added to patients showing increased blood pressure during the study. However, the ARB dosage remained unchanged. No diuretics were used in the study. Target blood pressure was below 130/80 mmHg.

All patients received routine diet and nutrition guidance to correctly record diet diaries (including food types and quantities), and regular outpatient follow-up was performed. During the study period, patients were visited by assigned investigators, who distributed medications, recovered medications, and exchanged salt bags to ensure patient compliance and safety.

### Biomarker assessment

The follow-up visits occurred at 0, 4, 8, and 12 weeks after treatment initiation. During each follow-up visit, blood pressure measurements and laboratory tests (blood and urine) were performed. Self-reported dietary intake and adverse events were documented. During visits at 0 and 12 weeks, electrocardiogram (ECG) and renal ultrasound were also performed as a safety assessment.

The primary endpoint was 24-h urine protein at the end of the intervention in the intent-to-treat (ITT) set and per-protocol (PP) set. The secondary endpoint was eGFR at the end of the intervention and was analyzed in the PP set. Nutritional, metabolic, and inflammatory biomarkers including serum albumin (ALB), serum pre-albumin (PA), blood uric acid (UA), total cholesterol (TC), triglycerides (TG), low-density lipoprotein-cholesterol (LDL-C), and C-reactive protein (CRP) were checked regularly. Besides ECG and renal ultrasound, safety indicators including blood routine, liver function, serum creatinine (Scr), blood urea nitrogen (BUN), and blood potassium were monitored regularly. Adverse events such as gynecomastia and sexual dysfunction were also monitored. Other indicators included 24-h urinary sodium (24 h-UNa), urine potassium (UK), blood pressure, and estimated dietary protein intake (eDPI) [34]. 24 h-UNa were monitored at 0, 4, 8, and 12 weeks.

### Statistical analysis

The last observation carried forward (LOCF) and a regression-based multiple imputation procedure were used to manage the missing data. Data were analyzed with SPSS 19.0 (IBM Corp, Armonk, NY, USA). Continuous variables with a normal distribution were presented as means ± standard deviation (SD) and compared by one-way analysis of variance (ANOVA) among three groups. Paired t-test was used to compare results between two time points in each group. Continuous variables with skewed distribution were presented as median and interquartile range and were compared by the Kruskal-Wallis test among three groups. The Wilcoxon rank-sum test was used to compare results between two time points in each group. Categorical variables were presented as frequency and percentage and were compared by Pearson Chi-squared test. *P* < 0.05 was considered statistically significant.

## Results

### Patient disposition and characteristics

**Fig. 1:**
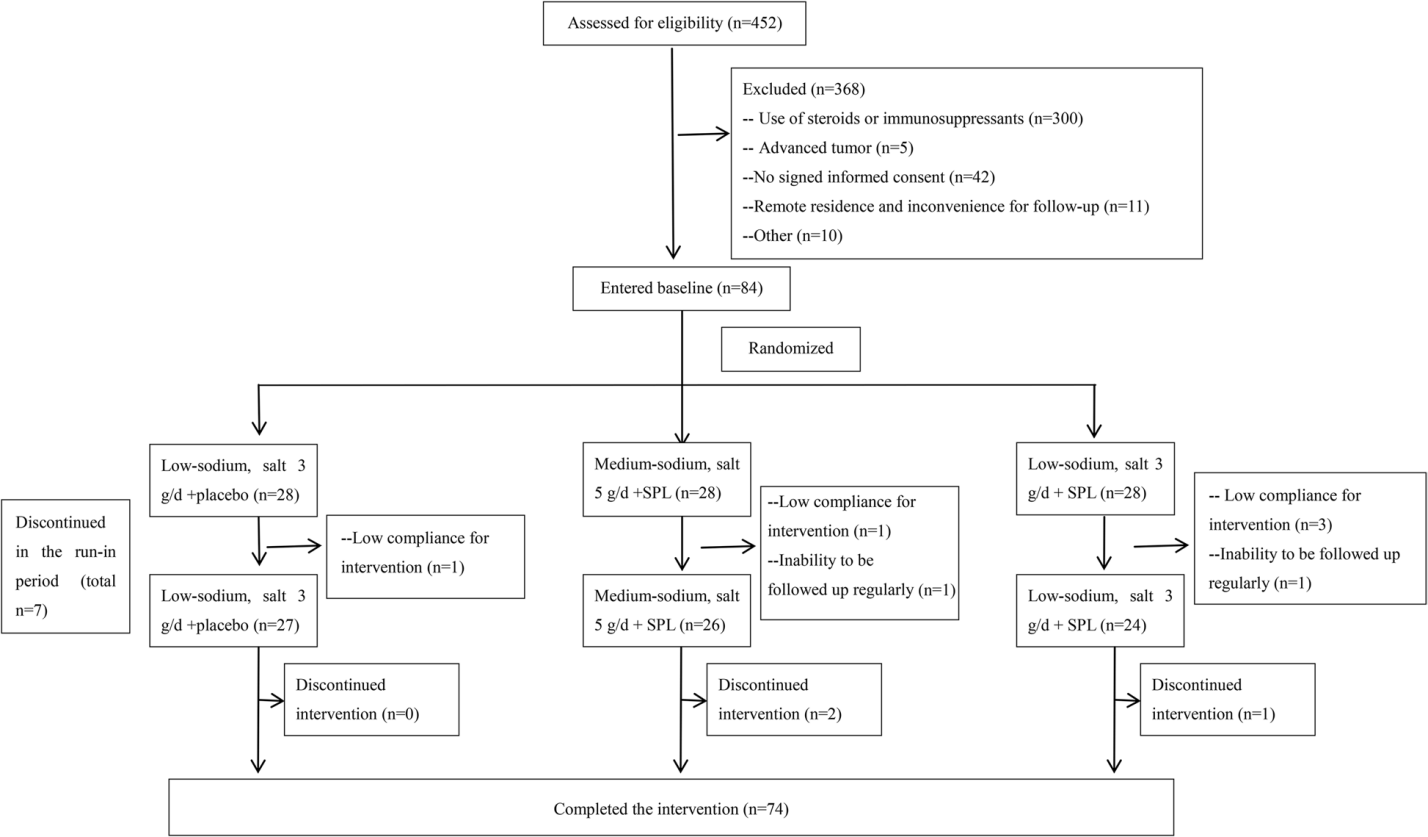
Patient flow chart

We assessed 452 patients for eligibility. Of these, 368 were excluded because of no signed informed consent (*n* = 42), current steroid or immunosuppressant therapy (*n* = 300), advanced tumors (*n* = 5), remote residence inconvenient for review (*n* = 11), and other reasons (*n* = 10). A total of 84 patients were randomized into three groups with 28 patients per group and started the run-in period, with seven withdrawing. Finally, 77 patients started the intervention period, including 27, 26, and 24 in the low-sodium+placebo, medium-sodium+SPL, and low-sodium+SPL groups, respectively. During the intervention period, two patients and one patient withdrew from the medium-sodium+SPL group and low-sodium+SPL group, respectively. A total of 74 patients completed the 12 weeks of intervention (Fig. 1).

These 74 patients consisted of 39 males and 35 females. Their diagnoses included: 40 patients with IgA nephropathy, two patients with membranous nephropathy, two patients with mesangial proliferative glomerulonephritis, one patient with minimal change disease, and 29 patients with chronic glomerulonephritis not otherwise specified. Sixty-three patients were stage 1–2 CKD, and 11 patients were stage 3a CKD. There were two patients in the low-sodium+SPL group who had diabetes (one patient had a history of diabetes for five years and another for three years). There were no significant differences in sex, age, body mass index (BMI), 24-h urine protein, eGFR, Scr, BUN, blood pressure, nutritional, metabolic, inflammatory, and other biomarkers among the three groups at baseline (Table 1).

**Table 1:** Baseline characteristics

Parameters	Low-sodium+placebo (*n* = 27)	Medium-sodium+SPL (*n* = 24)	Low-sodium+SPL (*n* = 23)	*P*
Male, N (%)	14 (52%)	9 (38%)	16 (70%)	0.763
Age (years)	42.44 ± 11.52	39.71 ± 9.68	43.00 ± 12.65	0.563
CKD stages, N (%)				0.967
Stage 1	16 (59%)	15 (62%)	13 (57%)	
Stage 2	7 (26%)	5 (21%)	7 (30%)	
Stage 3a	4 (15%)	4 (17%)	3 (13%)	
Renal diagnosis, N (%)				0.791
IgA nephropathy	13 (48%)	14 (58%)	13 (57%)	
MsPGN	0	1 (4%)	1 (4%)	
MN	0	1 (4%)	1 (4%)	
MCD	1 (4%)	0	0	
No renal biopsy	13 (48%)	8 (33%)	8 (35%)	
Antihypertensive drugs, N (%)				
ARB	20 (74%)	19 (79%)	20 (87%)	0.548
CCB	3 (11%)	2 (8%)	3 (13%)	0.901
β-blocker	0	1	0	
BMI (kg/m^2^)	24.16 ± 3.42	23.50 ± 3.60	23.45 ± 3.65	0.728
TC (mmol/L)	4.57 ± 0.86	4.56 ± 1.07	4.54 ± 0.77	0.993
TG (mmol/L)	1.38 ± 0.62	2.30 ± 2.06	1.75 ± 0.90	0.053
LDL-C (mmol/L)	2.57 ± 0.56	2.83 ± 0.78	2.76 ± 0.70	0.364
SBP (mmHg)	118.15 ± 9.81	119.29 ± 11.51	122.30 ± 11.85	0.403
DBP (mmHg)	72.30 ± 7.31	72.88 ± 9.13	72.87 ± 10.46	0.965
Blood potassium (mmol/L)	4.34 ± 0.39	4.22 ± 0.39	4.23 ± 0.40	0.476
UNa (mmol/d)	138.48 ± 60.92	157.70 ± 70.00	154.68 ± 87.55	0.598
UK (mmol/d)	54.29 ± 17.61	51.25 ± 13.57	51.31 ± 11.32	0.700
24-h urine protein (g/d)	0.37 (0.23, 0.67)	0.41 (0.32, 0.64)	0.35 (0.28, 0.56)	0.482
24-h urine creatinine (mmol/d)	10.91 (9.03,13.03)	9.41 (7.13,11.91)	11.20 (8.99,13.14)	0.128
24-h urine protein to creatinine ratio (g/mol)	30.22 (19.00,69.46)	50.88 (27.91,73.03)	34.89 (26.60,52.94)	0.342
eGFR (ml/min/1.73m^2^)	93.15 ± 30.94	98.18 ± 30.75	94.48 ± 27.11	0.826
Scr (μmol/L)	81.66 ± 28.61	76.42 ± 30.90	83.43 ± 28.73	0.694
BUN (mmol/L)	6.02 ± 1.62	5.66 ± 1.82	5.78 ± 2.03	0.767
Alb (g/L)	43.66 ± 2.26	42.78 ± 2.71	42.77 ± 2.64	0.356
PA (g/L)	0.33 ± 0.07	0.34 ± 0.06	0.35 ± 0.09	0.622
UA (μmol/L)	374.90 ± 75.84	360.29 ± 83.86	378.09 ± 75.51	0.705
CRP (mg/L)	1.76 ± 1.98	1.26 ± 1.01	1.51 ± 1.56	0.547
eDPI (g/kg/d)	1.06 ± 0.21	1.00 ± 0.26	1.08 ± 0.23	0.465

Note: Data at the end of the run-in period were taken as baseline values

*SPL* spironolactone, *MsPGN* mesangial proliferative glomerulonephritis, *MN* membranous nephropathy, *MCD* minimal change disease, *ARB* angiotensin II receptor blocker, *CCB* calcium channel blocker, *BMI* body mass index, *TC* total cholesterol, *TG* triglycerides, *LDL-C* low density lipoprotein-cholesterol, *SBP* systolic blood pressure, *DBP* diastolic blood pressure, *UNa* urine sodium, *UK* urine potassium, *eGFR* estimated glomerular filtration rate, *Scr* serum creatinine, *BUN* blood urea nitrogen, *Alb* albumin, *PA* pre-albumin, *UA* uric acid, *CRP* C-reactive protein, *eDPI* estimated daily protein intake

As for the salt levels of patients in this study, the median (range) of 24 h-UNa levels at 4, 8, 12 weeks fluctuated between 115 (93,150) mmol/d and 125 (91,156) mmol/d, 145 (110,217) mmol/d and 153 (117,190) mmol/d, and 114 (86,173) mmol/d and 126 (94,169) mmol/d in the low-sodium+placebo, medium-sodium+SPL, and low-sodium+SPL groups, respectively. Supplementary Table S1 shows that there were no differences in 24 h-UNa among the three groups at baseline (*P* = 0.598), but that the 24 h-UNA was significantly higher in the medium-sodium+SPL group compared with the low-sodium+placebo (*P* = 0.024) and low-sodium+SPL group (*P* = 0.007). Supplementary Table S2 shows that there were no differences in Una/Cr among the three groups at baseline (*P* = 0.303), but that the UNA/Cr was significantly higher in the medium-sodium+SPL group compared with the low-sodium+placebo (*P* = 0.011) and low-sodium+SPL group (*P* = 0.006).

### Efficacy

After 12 weeks of intervention, as the primary endpoint (ITT set), 24-h urine protein decreased from 0.37 (0.23, 0.70) to 0.23 (0.16, 0.51) in the low-sodium+placebo group (*P* = 0.004). The 24-h urine protein decreased from 0.44 (0.33, 0.71) to 0.29 (0.17, 0.50) in the medium-sodium+SPL group (*P* = 0.020). The 24-h urine protein decreased from 0.35 (0.26, 0.73) to 0.31 (0.22, 0.60) in the low-sodium+SPL group (*P* = 0.013). There were no significant differences among the three groups in 24-h urine protein amount changes after intervention from pre-treatment values (*P* = 0.760), as shown in Table 2.

**Table 2:** Clinical parameters before and after intervention (ITT set)

Parameters		Low-sodium+placebo (*n* = 28)	Medium-sodium+SPL (*n* = 28)	Low-sodium+SPL (*n* = 28)	*P among groups*
Primary endpoint 24-h urine protein (g/d)	Before intervention	0.37 (0.23,0.70)	0.44 (0.33,0.71)	0.35 (0.26,0.73)	0.365
	At 12 weeks	0.23 (0.16,0.51)	0.29 (0.17,0.50)	0.31 (0.22,0.60)	0.791
	Change between pre- and post- intervention	−0.06 (− 0.24,0)	−0.17 (− 0.25,-0.01)	− 0.12 (− 0.27,0.05)	0.760
	*P within group*	0.004	0.020	0.013	
24-h urine protein to creatinine ratio (g/mol)	Before intervention	30.54 (18.83,77.38)	54.00 (28.77, 81.30)	33.12 (25.90, 58.68)	0.248
	At 12 weeks	25.40 (14.12,50.71)	24.72 (18.49,44.10)	29.20 (15.69,62.39)	0.949
	Change between pre- and post- intervention	−3.72 (−16.42,1.04)	−15.85 (−41.40,-3.27)	−11.70 (−22.88,10.92)	0.118
	*P within group*	0.012	0.011	0.026	

Note: Data are mean:standard deviation or median (interquartile range)

*eGFR* estimated glomerular filtration rate

In the low-sodium+placebo group, the 24-h urine protein to creatine ratio was decreased from 30.54 (18.83, 77.38) to 25.40 (14.12, 50.71) (*P* = 0.012). Meanwhile, the 24-h urine protein to creatine ratios were decreased from 54.00 (28.77, 81.30) to 24.72 (18.49, 44.10), and from 33.12 (25.90, 58.68) to 29.20 (15.69, 62.39) in the medium-sodium+SPL (*P* = 0.011) and low-sodium+SPL (*P* = 0.026) groups, respectively. As for the changes pre- and post-intervention, there were no differences among the three groups (*P* = 0.118). The secondary endpoint eGFR also showed no significant differences among the three groups. In addition, the results of the 24-h urine protein by using PP set analysis was similar to the ITT set (PP set; Table 3).

**Table 3:** Clinical parameters before and after intervention (PP set)

Parameters		Low-sodium+placebo (*n* = 27)	Medium-sodium+SPL (*n* = 24)	Low-sodium+SPL (*n* = 23)	*P among groups*
Primary endpoint 24-h urine protein (g/d)	Before intervention	0.37 (0.23,0.67)	0.41 (0.32,0.64)	0.35 (0.28,0.56)	0.482
	At 12 weeks	0.21 (0.16,0.50)	0.27 (0.15,0.41)	0.30 (0.17,0.60)	0.827
	Change between pre- and post- intervention	−0.06 (−0.24,0)	−0.17 (−0.25,-0.01)	−0.12 (− 0.27,0.05)	0.721
	*P within the group*	0.004	0.009	0.021	
24 h urine protein to creatinine ratio (g/mol)	Before intervention	30.22 (19.00,69.46)	50.88 (27.91,73.03)	34.89 (26.60,52.94)	0.342
	At 12 weeks	24.62 (14.02,48.32)	23.77 (17.36,40.31)	30.00 (15.49,56.97)	0.957
	Change between pre- and post- intervention	−3.72 (−16.42,1.04)	−15.85 (−41.40,-3.27)	−11.70 (−22.88,10.92)	0.112
	*P within the group*	0.030	0.012	0.046	
Secondary endpoint eGFR (ml/min/1.73m^2^)	Before intervention	93.15 ± 30.94	98.18 ± 30.75	94.48 ± 27.11	0.826
	12 weeks	96.56 ± 31.20	93.50 ± 25.36	94.35 ± 28.99	0.925
	Change between pre- and post-intervention [95% confidence interval]	3.41 [−0.57,7.39]	−4.68 [−12.10,2.74]	−0.13 [−3.90,3.64]	0.082
	*P within the group*	0.090	0.205	0.944	

Note: Data are mean:standard deviation or median (interquartile range)

*eGFR* estimated glomerular filtration rate

### Safety and other indexes

During the intervention period of this clinical trial, hyperkalemia was not observed in any of the three groups. Average blood potassium levels ranged 4.26 ± 0.28 mmol/L in the low-sodium+placebo group, 4.29 ± 0.26 mmol/L in the medium-sodium+SPL group and 4.35 ± 0.32 mmol/L in the low-sodium+SPL group (*P* > 0.05). However, three patients (one patient with stage 2 CKD and two patients with stage 3a CKD, respectively) with increased blood potassium levels were observed in the low-sodium+SPL group. The range of blood potassium increase was 5.18–5.24 mmol/L. Dietary reports revealed that these three patients ate high-potassium foods. Their blood potassium decreased to 4.46–4.87 mmol/L after dietary guidance was provided without the need for any medication adjustments.

No gynecomastia or sexual dysfunction was observed in patients in the medium-sodium+SPL group and low-sodium+SPL group. No significant abnormal changes in blood routine, liver function, ECG parameters, and other routine safety indicators were observed in any patient. In addition, no progressive renal dysfunction (eGFR decreased by 30%) or all-cause death was observed during the study.

During the intervention period, nutritional, metabolic, and inflammatory markers, as well as blood pressure, were stable in all three groups. ALB levels in the low-sodium+placebo group were decreased after 12 weeks of intervention, but all fell within the normal range. TC and LDL-C levels in the low-sodium+placebo group decreased significantly (both *p* = 0.001), while LDL-C level in the low-sodium+placebo group after the intervention was significantly lower than the other two groups (both *p* < 0.05). The SBP and DBP all showed a decrease after intervention with no significant statistical difference among the three groups (Table 4).

**Table 4:** Other indicators before and after intervention

Parameters		Low-sodium+placebo (*n* = 27)	Medium-sodium+SPL (*n* = 24)	Low-sodium+SPL (*n* = 23)	*P*
ALB (g/L)	Before Intervention	43.66 ± 2.26	42.78 ± 2.71	42.77 ± 2.64	0.356
	After Intervention	42.60 ± 2.75	43.07 ± 1.97	43.12 ± 2.79	0.725
	*P*	0.018	0.677	0.401	
PA (g/L)	Before Intervention	0.33 ± 0.07	0.34 ± 0.06	0.35 ± 0.09	0.622
	After Intervention	0.33 ± 0.06	0.33 ± 0.06	0.35 ± 0.08	0.317
	*P*	0.824	0.214	0.843	
eDPI (g/kg/d)	Before Intervention	1.06 ± 0.21	1.00 ± 0.26	1.08 ± 0.23	0.465
	After Intervention	1.02 ± 0.25	1.05 ± 0.16	1.09 ± 0.17	0.385
	*P*	0.377	0.413	0.770	
Scr (μmol/L)	Before Intervention	81.66 ± 28.61	76.42 ± 30.90	83.43 ± 28.73	0.694
	After Intervention	77.68 ± 23.50	77.42 ± 29.20	83.96 ± 30.40	0.655
	*P*	0.020	0.696	0.780	
BUN (mmol/L)	Before Intervention	6.02 ± 1.62	5.66 ± 1.82	5.78 ± 2.03	0.767
	After Intervention	5.73 ± 2.07	5.97 ± 1.30	6.21 ± 2.20	0.666
	*P*	0.206	0.315	0.055	
UA (μmol/L)	Before Intervention	374.90 ± 75.84	360.29 ± 83.86	378.09 ± 75.51	0.705
	After Intervention	374.38 ± 82.16	385.08 ± 76.40	365.13 ± 88.35	0.709
	*P*	0.964	0.159	0.457	
TC (mmol/L)	Before Intervention	4.57 ± 0.86	4.56 ± 1.07	4.54 ± 0.77	0.993
	After Intervention	4.15 ± 0.73	4.68 ± 0.95	4.50 ± 0.93	0.092
	*P*	0.001	0.668	0.824	
TG (mmol/L)	Before Intervention	1.38 ± 0.62	2.30 ± 2.06	1.75 ± 0.90	0.053
	After Intervention	1.25 ± 0.60	1.68 ± 0.94	1.72 ± 0.76	0.060
	*P*	0.201	0.068	0.852	
LDL-C (mmol/L)	Before Intervention	2.57 ± 0.56	2.83 ± 0.78	2.76 ± 0.70	0.364
	After Intervention	2.19 ± 0.59^a)、b)^	2.82 ± 0.75	2.59 ± 0.71	0.006
	*P*	0.001	0.881	0.249	
CRP (mg/L)	Before Intervention	1.76 ± 1.98	1.26 ± 1.01	1.51 ± 1.56	0.547
	After Intervention	1.54 ± 1.86	1.42 ± 1.15	1.67 ± 2.30	0.890
	*P*	0.588	0.564	0.613	
SBP (mmHg)	Before Intervention	118.15 ± 9.81	119.29 ± 11.51	122.30 ± 11.85	0.403
	After Intervention	116.52 ± 9.69	113.46 ± 11.98	117.65 ± 9.73	0.367
	*P*	0.413	0.003	0.086	
DBP (mmHg)	Before Intervention	72.30 ± 7.31	72.88 ± 9.13	72.87 ± 10.46	0.965
	After Intervention	71.52 ± 7.76	69.42 ± 8.12	70.39 ± 9.17	0.668
	*P*	0.582	0.029	0.209	

a) *P* < 0.05, compared with medium-sodium (5 g/d salt) + SPL

b) *P *< 0.05, compared with low-sodium (3 g/d salt) + SPL

Data are mean ± standard deviation. *ALB* albumin, *PA* pre-albumin, *eDPI* estimated daily protein intake, *Scr* serum creatinine, *BUN* blood urea nitrogen, *UA* uric acid, *CRP* C-reactive protein, *SBP* systolic blood pressure, *DBP* diastolic blood pressure

## Discussion

The key observations of this study are as follows. First, no further benefit exists from the addition of SPL in patients with a low sodium diet (3.0 g/d salt). Second, small doses of SPL may benefit patients with poor sodium restriction.

Under the condition of basic treatment, 24-h urine protein to creatine ratios were decreased significantly in all three groups of ITT or PP set after 12 weeks of intervention, but there were no significant differences among the three groups (*P* > 0.05). These results suggested that sodium control and/or SPL might have renal protective effects in patients with stage 1-3a CKD.

Few clinical studies have assessed the relationship between salt intake and MRA administration, and a literature review found no related randomized controlled trials. A post hoc analysis of MRA efficacy stratified by urinary sodium excretion of the Eplerenone Combination Versus Conventional Agents to Lower Blood Pressure on Urinary Antialbuminuric Treatment Effect (EVALUATE) trial was recently conducted [35]. In this study, the population included patients with CKD and hypertension but without diabetes, and the basic treatment was ACEI and/or ARB. The results showed that eplerenone-treated patients in the highest sodium excretion tertile exhibited significantly greater reduction in urinary albumin to creatinine ratio (UACR) compared with the placebo subjects in the same tertile (− 22.5% vs. + 21.8%, *p* = 0.02). While this disparity was not observed neither in the lowest tertile (− 10.2% vs. -0.84%, *p* = 0.65) nor in the middle tertile (− 19.5% vs. + 9.5%, *p* = 0.22). These authors concluded that the therapeutic effect of MRA is related to salt intake. In patients with high urinary sodium, the antiproteinuric effect of MRA was more significant than that of a placebo, while in patients with low urinary sodium, the antiproteinuric effect was not significantly different [35]. This conclusion corroborated our findings. The possible mechanism is as follows. Salt loading could enhance the renal MR activation pathway by activating Rac1 without increasing circulating aldosterone, causing hypertension and renal damage; meanwhile, MRA blocks this pathway, inhibits high-salt intake associated renal injury, overcomes salt-associated resistance to routine RAS blockade treatment, and plays a role of renal protection [36]. Similar results have been found in patients with refractory hypertension. The higher the salt intake level, the greater the blood pressure response to SPL treatment, suggesting that MRA could antagonize the increase of blood pressure caused by high salt intake [37].

As a simple and cost-effective treatment, dietary salt restriction delays CKD progression. However, patients with CKD show poor adherence to a low-salt diet in their daily life. Data showed that average 24 h urine sodium excretion in patients with CKD ranges between 144 and 200 mmol/d [3, 38–41]. It is challenging to achieve salt restriction even in clinical trials. Average daily salt consumption is 9–13 g/d in reported CKD clinical studies up to now [42]. Adding SPL to the treatment regimen may be a potential new strategy in patients with CKD that are unable to achieve the ideal salt restriction goal.

Hyperkalemia is the main adverse side effect in patients taking MRA, especially in combination with ACEI and/or ARB. Previous studies have shown that the risk of hyperkalemia after treatment with MRA combined with ACEI and/or ARB is elevated compared with ACEI and/or ARB alone [22, 23, 43–46]. Epstein et al. carefully evaluated hyperkalemia incidence in 268 patients, and blood potassium levels showed no significant change in MRA combined with ACEI compared to ACEI alone [47]. During the 12 week intervention period in this study, there was no hyperkalemia in medium-sodium+SPL and low-sodium+SPL groups. Even in patients with ESRD, previous research has suggested that treatment is safe provided that the medication indication is accurate and strict monitoring parameters are applied [48].

Gynecomastia resulting from the combination of aldosterone with androgen and progesterone receptors is often reported as a side effect of SPL, thereby limiting its clinical usefulness. In the RALES study, gynecomastia incidence in patients treated with SPL was 9% versus 1% in the placebo arm [49]. The use of the selective MRA EPL could reduce these risks and other side effects [50]. In addition, finerenone, a nonsteroidal, selective mineralocorticoid receptor antagonist, reduced the risks of CKD progression and cardiovascular events in patients with CKD and type 2 diabetes compared with placebo in the FIDELIO-DKD trial; meanwhile, the overall rates of adverse events were similar in both groups [51]. In this study, the non-selective MRA SPL was administered at 20 mg/day, and no significant adverse events were found in the medium-sodium+SPL and low-sodium+SPL groups throughout the study.

Several limitations in this clinical trial are noted as follows. First, although this was a prospective, randomized controlled study (medication administration was performed in a blinded manner), salt intake could not be blinded. Secondly, based on ethical principles, a high salt group (oral salt tablet) was not established in this study due to the proven high salt-associated renal damage. Thirdly, low-dose ARB was administered to most patients, and the independent effect of salt restriction alone or salt restriction combined with MRA could not be explained. Fourthly, this was a single-center pilot study, with a relatively small sample size, relatively normal kidney function, and short follow-up, which limited the evaluation of the efficacy and long-term safety profile. Fifthly, long-term data for various endpoints were not obtained. Based on these limitations, larger multicenter clinical trials with longer follow-up are required to further evaluate the effects and safety of sodium restriction and MRAs.

## Conclusion

Under low sodium intake conditions, ARB combined with SPL has no additional benefits on lowering urine protein compared with ARB alone. It is unnecessary to add on SPL for patients with satisfying sodium restriction, thus avoiding the adverse events of SPL. In patients with poor sodium restriction, supplementation of low-dose SPL might show a therapeutic benefit on lowering 24-h urine protein. The combination of short-term low-dose SPL and ARB is safe in patients with stage 1-3a CKD, but blood potassium must be carefully monitored.

## Supplementary Information

**Additional file 1: Supplementary Table 1.** Urine sodium excretion among the three groups at 12 weeks.

**Additional file 2: Supplementary Table 2.** Urinary sodium/creatinine ratio among the three groups at 12 weeks.

## Data Availability

The datasets used and/or analyzed during the current study are available from the corresponding author on reasonable request.

## Abbreviations

CKD: Chronic kidney disease
SPL: Spironolactone
eGFR: Estimated glomerular filtration rate
ESRD: End-stage renal disease
TGF: Transforming growth factor
RAAS: Renin-angiotensin-aldosterone system
ACEIs: Angiotensin-converting enzyme inhibitors
ARBs: Angiotensin II receptor blockers
NSAIDs: Nonsteroidal anti-inflammatory medications
ALB: Albumin
PA: Pre-albumin
UA: Uric acid
TC: Total cholesterol
TG: Triglycerides
LDL-C: low density lipoprotein-cholesterol
CRP: C-reactive protein
Scr: serum creatinine
BUN: Blood urea nitrogen
24 h-UNa: 24-h urinary sodium
UK: Urine potassium
eDPI: Estimated dietary protein intake
SD: Standard deviation
ANOVA: Analysis of variance
