# Supplementary material for: Efficacy and safety of a low-sodium diet and spironolactone in patients with stage 1-3a chronic kidney disease: a pilot study
Source: BMC Nephrol. 2022 Mar 5;23:95. doi: 10.1186/s12882-022-02711-z (PMC8897863; doi:10.1186/s12882-022-02711-z)
Supplement: Supplementary file 1 — Additional file 1: Supplementary Table 1. Urine sodium excretion among the three groups at 12 weeks. [file 12882_2022_2711_MOESM1_ESM.docx]

**Supplementary Table 1.** Urine sodium excretion among the three groups at 12 weeks

| Parameters | | Low-sodium+placebo (n=27) | Medium-sodium+SPL (n=24) | Low-sodium+SPL (n=23) | *P among groups* |
| --- | --- | --- | --- | --- | --- |
| UNa | 0 week | 138.48±60.92 | 157.70±70.00 | 154.68±87.55 | 0.598 |
|  | 12 weeks | 131.89±48.15 | 165.46±60.60^※△^ | 123.38±46.02 | 0.016 |

^※^Compared with Low-sodium+placebo, P=0.024; ^△^Compared with Low-sodium+SPL, P=0.007. P < 0.05 is considered significant.
