# Supplementary material for: Efficacy and safety of a low-sodium diet and spironolactone in patients with stage 1-3a chronic kidney disease: a pilot study
Source: BMC Nephrol. 2022 Mar 5;23:95. doi: 10.1186/s12882-022-02711-z (PMC8897863; doi:10.1186/s12882-022-02711-z)
Supplement: Supplementary file 2 — Additional file 2: Supplementary Table 2. Urinary sodium/creatinine ratio among the three groups at 12 weeks. [file 12882_2022_2711_MOESM2_ESM.docx]

**Supplementary Table 2.** Urinary sodium/creatinine ratio among the three groups at 12 weeks

| Parameters | | Low-sodium+placebo (n=27) | Medium-sodium+SPL (n=24) | Low-sodium+SPL (n=23) | *P among groups* |
| --- | --- | --- | --- | --- | --- |
| UNa/Cr | 0 week | 10.64 (8.23,13.96) | 14.38 (9.89,19.88) | 13.26 (9.23,19.12) | 0.303 |
|  | 12week | 12.01(10.15,14.83) | 14.45(11.66,18.38)^※△^ | 12.18(8.12,15.23) | 0.008 |

^※^Compared with Low-sodium+placebo, P=0.011; ^△^Compared with Low-sodium+SPL, P=0.006. P < 0.017 is considered significant.
